# Editorial: Community series in renal fibrosis and renal transplantation, volume II

**DOI:** 10.3389/fimmu.2026.1951269

**Published:** 2026-09-03

**Authors:** Cheng Yang

**Affiliations:** 1 Department of Kidney Transplantation, Zhongshan Hospital, Fudan University, Shanghai, China; 2 Shanghai Key Laboratory of Organ Transplantation, Shanghai, China; 3 Zhangjiang Institute of Fudan University, Shanghai, China; 4 Shanghai Institute of Medical Imaging, Shanghai, China

**Keywords:** biomarkers, cell metabolism, cell signal, environmental exposures, epigenetics, microenvironment, renal fibrosis, renal transplantation

## Introduction

Renal fibrosis represents the final common pathway of virtually all progressive chronic kidney diseases (CKD) and serves as a primary driver of long-term renal allograft failure. Building upon the foundational insights established in Volume I, this second volume of the “Community Series in Renal Fibrosis and Renal Transplantation” brings together 17 high-impact contributions—comprising 13 original research articles and 4 comprehensive reviews—that further unravel the cellular, molecular, epigenetic, and metabolic networks governing fibrogenesis and graft dysfunction.

## Molecular mechanisms, epigenetic regulation, and intracellular signaling

Understanding intracellular cascades and epigenetic modifications is crucial for identifying novel therapeutic targets to halt fibrotic remodeling. Epigenetic regulation via m6A RNA modification plays a key role in CKD progression; Xu et al. demonstrated that METTL3-mediated m6A modification stabilizes NLRC5 mRNA, exacerbating renal fibrosis by suppressing the antioxidant Keap1/Nrf2/ARE signaling pathway. Therapeutic targeting of metabolic stress pathways also shows significant promise; Lai et al. reported that TB001, a novel GCGR/GLP-1R dual-agonist, attenuates tubular epithelial-mesenchymal transition and interstitial fibrosis by inhibiting PERK-mediated endoplasmic reticulum stress. In acute injury settings, Ma et al. uncovered that Laptm4a exacerbates renal ischemia-reperfusion injury (IRI) by suppressing the protective UNC5B-AKT/mTOR axis. Furthermore, in chronic allograft injury, Deng et al. revealed that miR-204-5p attenuates early inflammatory infiltration in chronic renal allograft dysfunction (CRAD) by directly targeting IL-11 and inhibiting ERK1/2-NF-κB signaling.

## Immune microenvironment, multicellular crosstalk, and cellular heterogeneity

Renal fibrogenesis is actively regulated by dynamic multicellular interactions within the local immune microenvironment. Utilizing single-cell RNA sequencing, Wei et al. mapped the early immune landscape of renal allograft rejection, identifying a pro-inflammatory Isg15+ macrophage subset that drives T-cell activation through the Ccl3–Ccr5 axis—a pathway effectively blunted by the FDA-approved Ccr5 blocker Maraviroc. Highlighting novel stromal cell dynamics, Schütz et al. identified LOXL2 as a marker for a distinct subset of inflammation-associated myofibroblasts that rapidly expand during rejection and serve as long-term predictors of graft dysfunction. Providing a comprehensive overview of cellular networks, Ding et al. reviewed the complex crosstalk among immune cells, renal epithelial cells, and stromal cells during interstitial fibrosis, identifying key regulatory nodes for precision intervention. Complementing innate immunity insights, Wang et al. reviewed the dual paradigm of NLRP3 inflammasome signaling across IRI, rejection, and infection. In addition, exploring post-translational metabolic modifications, Yuan et al. integrated single-nucleus transcriptomics and machine learning to uncover five lactylation-related hub genes (IKZF1, PDLIM1, S100A11, STAT4, and SLC2A3) that strongly correlate with immune cell infiltration and allograft fibrosis.

## Biomarkers, molecular imaging, and microfluidic technologies

Developing non-invasive diagnostic tools and real-time monitoring modalities is essential for early clinical intervention. Overcoming the limitations of invasive tissue biopsies, Wang et al. engineered a near-infrared fluorescent GLUT1 probe (XJYZ) specific to M1 macrophages, enabling early, non-invasive, dynamic visualization of T cell-mediated rejection. Evaluating circulating biomarkers, Wang et al. established that plasma sADAM10 serves as a context-dependent biomarker independently associated with eGFR in CKD and infection risk in kidney transplant recipients. For perioperative management, Yan et al. showed that combining post-transplant serum Tenascin-C levels and biopsy staining scores significantly enhances the predictive accuracy for delayed graft function (DGF). Synthesizing engineering advances, Liu et al. reviewed microfluidic organ-on-a-chip models, biosensors, and nanodelivery systems that bridge mechanistic discovery with precision nephrology.

## Clinical risk stratification, environmental exposures, and novel transplant paradigms

Translating mechanistic concepts into clinical management requires refined risk assessment and environmental awareness. In pediatric transplantation, Li et al. analyzed 535 recipients, demonstrating that a Banff Chronicity Index (CI) ≥ 4 serves as an independent predictor of long-term functional decline, whereas the Activity Index primarily reflects short-term impairment. Addressing strategies to expand donor pools, Chen et al. evaluated ABO-incompatible living donor kidney transplantation in China, demonstrating that modern desensitization protocols yield short- to mid-term patient and graft survival rates comparable to ABO-compatible transplants. Extending beyond allotransplantation into xenogeneic models, Jiang et al. investigated acute humoral xenograft rejection (AHXR) in GTKO/β4GalNT2KO-based pig-to-rhesus monkey renal xenotransplantation, demonstrating that elicited antibodies against unidentified “neoantigens” play a key role in driving AHXR and graft pathology. Finally, Li et al. reviewed the impact of environmental heavy metal exposure on graft failure risk, emphasizing oxidative stress pathways and proposing biomonitoring alongside targeted antioxidant therapies.

## Conclusion

In summary, Volume II of this Community Series highlights major advances across epigenetic regulation, immune cell crosstalk, novel biomarkers, bio-imaging, and clinical/xenogeneic transplant paradigms. Collectively, these 17 articles advance our mechanistic understanding of renal fibrogenesis and offer promising strategies to enhance renal allograft survival and CKD patient outcomes.

